# Altered functional connectivity of the thalamus in primary angle-closure glaucoma patients: A resting-state fMRI study

**DOI:** 10.3389/fneur.2022.1015758

**Published:** 2022-10-06

**Authors:** Yuanyuan Wang, Linglong Chen, Fengqin Cai, Junwei Gao, Feng Ouyang, Ye Chen, Mingxue Yin, Chengpeng Hua, Xianjun Zeng

**Affiliations:** ^1^Department of Radiology, The First Affiliated Hospital of Nanchang University, Nanchang, China; ^2^Department of Cardiology, The Second Affiliated Hospital of Nanchang University, Nanchang, China

**Keywords:** primary angle-closure glaucoma, thalamus, resting-state functional MRI, functional connectivity, visual impairment

## Abstract

**Background and objectives:**

Glaucoma is one of the leading irreversible causes of blindness worldwide, and previous studies have shown that there is abnormal functional connectivity (FC) in the visual cortex of glaucoma patients. The thalamus is a relay nucleus for visual signals; however, it is not yet clear how the FC of the thalamus is altered in glaucoma. This study investigated the alterations in thalamic FC in patients with primary angle-closure glaucoma (PACG) by using resting-state functional MRI (rs-fMRI). We hypothesized that PACG patients have abnormal FC between the thalamus and visual as well as extravisual brain regions.

**Methods:**

Clinically confirmed PACG patients and age- and gender-matched healthy controls (HCs) were evaluated by T1 anatomical and functional MRI on a 3 T scanner. Thirty-four PACG patients and 33 HCs were included in the rs-fMRI analysis. All PACG patients underwent complete ophthalmological examinations; included retinal nerve fiber layer thickness (RNFLT), intraocular pressure (IOP), average cup-to-disc ratio (A-C/D), and vertical cup-to-disc ratio (V-C/D). After the MRI data were preprocessed, the bilateral thalamus was chosen as the seed point; and the differences in resting-state FC between groups were evaluated. The brain regions that significantly differed between PACG patients and HCs were identified, and the correlations were then evaluated between the FC coefficients of these regions and clinical variables.

**Results:**

Compared with the HCs, the PACG patients showed decreased FC between the bilateral thalamus and right transverse temporal gyrus, between the bilateral thalamus and left anterior cingulate cortex, and between the left thalamus and left insula. Concurrently, increased FC was found between the bilateral thalamus and left superior frontal gyrus in PACG patients. The FC between the bilateral thalamus and left superior frontal gyrus was positively correlated with RNFLT and negatively correlated with the A-C/D and V-C/D. The FC between the left thalamus and left insula was negatively correlated with IOP.

**Conclusion:**

Extensive abnormal resting-state functional connections between the thalamus and visual and extravisual brain areas were found in PACG patients, and there were certain correlations with clinical variables, suggesting that abnormal thalamic FC plays an important role in the progression of PACG.

## Introduction

Glaucoma, a group of blinding eye diseases characterized by optic atrophy and visual field defects, is one of the major irreversible causes of blindness worldwide. Currently, there are ~76 million glaucoma patients in our country, and the number is expected to increase to 1.12 billion by 2040 (1). Primary glaucoma can be divided into primary angle-closure glaucoma (PACG) and primary open-angle glaucoma (POAG). Increased intraocular pressure (IOP) is the most important risk factor for glaucoma, and damage to retinal ganglion cells (RGCs) by elevated IOP is considered to a key factor in the pathogenesis of glaucoma (2, 3). However, RGCs are extensions of the central nervous system developmentally and anatomically. And many studies have shown that glaucoma is not only an eye disease but also a degenerative disease of the central nervous system (4–9).

Previous MRI studies mostly focused on POAG, which has a high incidence in Western countries, and found spontaneous functional connectivity changes in the primary visual cortex (V1) of POAG patients (10, 11). By contrast, in China and throughout Asia, the most common type of glaucoma is PACG (12), and glaucoma patients there suffer a higher prevalence of blindness than those in Western countries (13). Recently, resting-state functional MRI (rs-fMRI) has been increasingly used to explore intrinsic brain activity in glaucoma patients and has provided critical information in the search for pathological mechanisms (14, 15). Several recent studies have shown that PACG patients exhibit abnormalities in regional homogeneity (ReHo) (16, 17) and amplitude of low-frequency fluctuation (ALFF) (18, 19) in multiple brain regions. And Tong et al. (20) found that there is disturbed interhemispheric resting-state functional connectivity in the vision-related brain areas of individuals with PACG. In additional, our group also found that PACG patients showed brain dysfunction in visual and other regions and presented different spatial distributions of short-range and long-range functional connectivity density (FCD) (21).

As the largest mixed nucleus of gray and white matter in the deep part of the brain, the thalamus is involved in multiple functions, such as visual information transmission, cognition, emotion, sensation, and movement. The alternation of thalamus in the visual conduction pathway are the main impairment of central nervous system in glaucoma (22–24). Karlen et al. (25) demonstrated that early blindness showed alterations of thalamocortical connections. Reislev et al. (26) found that blind individuals showed thalamocortical connectivity and microstructural changes. And a recent animal experiment (27) suggested that changes in the thalamus may predate damage to RGCs. However, the literature on thalamic function in PACG patients remains scarce.

In this study, we searched for evidence of abnormal functional connectivity in the thalamus of PACG patients by examining resting-state functional connectivity (rs-FC), which focuses on statistical correlations between signals in the whole brain or within brain regions (28), showing great potential as a method to explore synchronous activity in the brain. The main purposes of this study were to explore the FC relationship between the thalamus and the whole brain of PACG patients, and relate the alterations in FC to clinical data by analyzing the correlation between clinical parameters and the significantly changed regions.

## Materials and methods

### Subjects

Forty right-handed PACG patients were recruited from the Ophthalmology Department of the First Affiliated Hospital of Nanchang University. The inclusion criteria for the PACG patients were as follows: (1) narrow anterior chamber angles in both eyes, confirmed clinically by gonioscopy and slit-lamp examination; (2) visual field defects associated with glaucoma, such as tubular vision and nasal hemianopia; (3) an optic nerve cup-to-disc ratio > 0.6, as determined by funduscopic examination; and (4) didn't received medical or surgical treatment for glaucoma. The exclusion criteria for the PACG patients were as follows: (1) the diagnosis of another type of glaucoma, such as POAG or secondary glaucoma; (2) the diagnosis of another ocular disease or an organic disorder affecting the visual pathway; (3) a history of brain trauma; (4) a history of underlying disease, such as hypertension or diabetes; (5) a history of surgical treatment for glaucoma; (6) incomplete data from MRI scans or clinical assessment; and (7) head movement exceeding 2.5 mm maximum displacement in the x, y, and/or z directions or 2.5° of angular rotation about any axis during the rs-fMRI examination. Ultimately, 34 PACG patients (16 males and 18 females) were included in this study (4 had incomplete clinical data, and two had head movement > 2.5 mm or > 2.5° during the rs-fMRI examination).

We recruited and selected 33 right-handed, age- and gender-matched healthy subjects to serve as healthy controls (HCs; 16 males and 17 females). The exclusion criteria for the HCs were as follows: (1) the diagnosis of an ocular disorder or other systemic disease; (2) severe nearsightedness or farsightedness; (3) contraindications for MRI, such as metal implants or claustrophobia; and (4) head movement exceeding 2.5 mm maximum displacement in the x, y, and/or z directions or 2.5° of angular rotation about any axis.

This study complied with the Declaration of Helsinki, and the Human Research Ethics Committee of the First Affiliated Hospital of Nanchang University approved the study protocol. Written informed consent was obtained from each participant prior to the study. Table 1 provides information on the demographics of the PACG patients and HCs.

**Table 1:** General clinical information for PACG patients and HCs.

**Condition**	**PACG**	**HC**	***P*-value**
Age (years)	53.12 ± 11.75	52.85 ± 10.61	0.922
Gender (male/female)	16/18	16/17	0.907
Duration of PACG (days) (range)	2–3,650	-	N/A
IOP (mmHg)	27.34 ± 9.24	-	N/A
RNFLT (μm)	87.77 ± 24.96	-	N/A
A-C/D	0.67 ± 0.15	-	N/A
V-C/D	0.64 ± 0.18	-	N/A
Mean VA (range)	0.53 ± 0.31	-	N/A
Head motion	0.064 (0.048, 0.089)	0.072 (0.055, 0.098)	0.243

PACG, primary angle-closure glaucoma; HCs, healthy controls; IOP, RNFLT, A-C/D, V-C/D, and VA are presented as the binocular mean values. IOP, intraocular pressure; RNFLT, retinal nerve fiber layer thickness; A-C/D, average cup-to-disc ratio; V-C/D, vertical cup-to-disc ratio; VA, visual acuity; N/A, not applicable.

### Data acquisition

The rs-fMRI data were acquired using a 3T MR scanner (Siemens, Erlangen, Germany) with an 8-channel phased-array head coil at the Department of Radiology of the First Affiliated Hospital, Nanchang University, China. All subjects were directed to sit for 10 min prior to their resting-state scans. Next, they were instructed to keep their eyes closed but to not fall asleep. The subjects were further instructed to not engage in any specific cognitive activity during data acquisition. Head movements and noise were suppressed using a suitable sponge mat and earplugs, respectively. Rs-fMRI data acquisition lasted for 8 min, and 240 resting-state volumes were acquired using the following parameters: repetition time (TR) = 2,000 ms; echo time (TE) = 40 ms; flip angle = 90°; field of view (FOV) = 240 mm × 240 mm; matrix =64 × 64; and slice thickness = 4 mm with a 1 mm gap. Each brain volume included 30 axial slices. High-resolution T1-weighted images for each subject were acquired using a 3D MRI sequence with the following parameters: TR = 1,900 ms; TE = 2.26 ms; flip angle = 9°; FOV= 240 mm × 240 mm; matrix = 256 × 256; number of sagittal slices = 176; and slice thickness = 1 mm.

### Data preprocessing

MRIcro software was used to check the resting-state fMRI data and discard any data of suboptimal quality. Rs-fMRI data were preprocessed with the Data Processing & Analysis for Brain Imaging (DPABI) toolbox (29), which was run in MATLAB 2018b (MathWorks, Natick, MA, United States). First, the first 10 time points were removed. Then, the remaining 230 volumes were corrected for slice timing and three-dimensional head motion. Two PACG patients were excluded because the maximum displacement in at least one direction (x, y, and/or z) was more than 2.0 mm; the angular rotation about some axis exceeded 2.0°; or the framewise displacement (FD), a relative displacement measure, exceeded 2.5 standard deviations for any of the 230 volumes during the entire fMRI scanning process. Then, all the functional data were spatially normalized to the Montreal Neurological Institute (MNI) template using non-linear transformation procedures in SPM12 (30), resampled to 3 mm × 3 mm × 3 mm voxels, and smoothed with a 6-mm full width at half maximum (FWHM) filter. Finally, a temporal filter (0.01–0.08 Hz) was utilized to suppress the effects of low-frequency drift and high-frequency noise. To further reduce the influence of confounding factors, a multiple regression method was performed to regress out interference, including the mean time series of all white matter and cerebrospinal fluid voxels, global signals and Friston's 24-parameter model of head motion (6 head motion parameters, six head motion parameters from one time point earlier, and the 12 corresponding squared items) (31–33).

### Seed-based functional connectivity analysis

We selected the bilateral thalamic region marked by the automated anatomical labeling (AAL) template in WFU_PickAtlas software as the region of interest (ROI) for FC analysis and extracted the time series of all voxels in the ROI as the time series of the ROI; the same was done for the whole brain. The time series of all voxels were subjected to Pearson correlation FC analysis to obtain an FC correlation coefficient map; Fisher's z transformation was then performed to align the data more closely with the normal distribution and facilitate statistical analysis.

### Clinical assessment

All patients underwent detailed ophthalmological examination. The state of the anterior chamber angle was determined by gonioscopy and slit-lamp examination. IOP was measured using a tonometer, while retinal nerve fiber layer thickness (RNFLT), the average cup-to-disc ratio (A-C/D) and the vertical cup-to-disc ratio (V-C/D) were evaluated by optical coherence tomography (Cirrus HD-OCT). In addition, the disease course and visual acuity (VA) were recorded.

### Statistical analysis

The chi-square test was used to compare the categorical variable of gender between groups, and an independent-samples *t*-test was used for measurement variables (age and clinical ophthalmology parameters); these tests were implemented in the Statistical Package for the Social Sciences (SPSS) version 24.0 (Chicago, IL, United States). When *p* < 0.05, the differences were considered significant.

For the rs-FC between each ROI and the remaining voxels of the brain, two-sample *t*-tests were conducted between the two groups to identify differences in spatial distribution. Then, analysis of covariance was applied to analyze intergroup differences, with gender and age as covariates, using DPABI software in MATLAB 2018b (MathWorks, Natick, MA, United States). The results were corrected using AlphaSim implemented in DPABI Viewer with p <0.05 and were reported using REST V1.84. The resulting z value maps were overlaid on the rendered views using BrainNet Viewer, and the locations of the brain regions with significant FC were reported using xjView software.

To examine whether there is a relationship between abnormal FC and clinical variables, we used the statistical software package SPSS 24.0 to conduct Pearson correlation analysis between the FC strength of different brain regions and the results of clinical scales; *P* < 0.05 was considered statistically significant.

## Results

### Demographic and clinical data

As shown in Table 1, the mean age of PACG patients was 53.12 ± 11.75 years, and the disease duration ranged from 2 days to 3,650 days. There were no significant differences between PACG patients and HCs in age, gender.

### Differences in resting-state functional connectivity between the two groups

**Figure 1:**
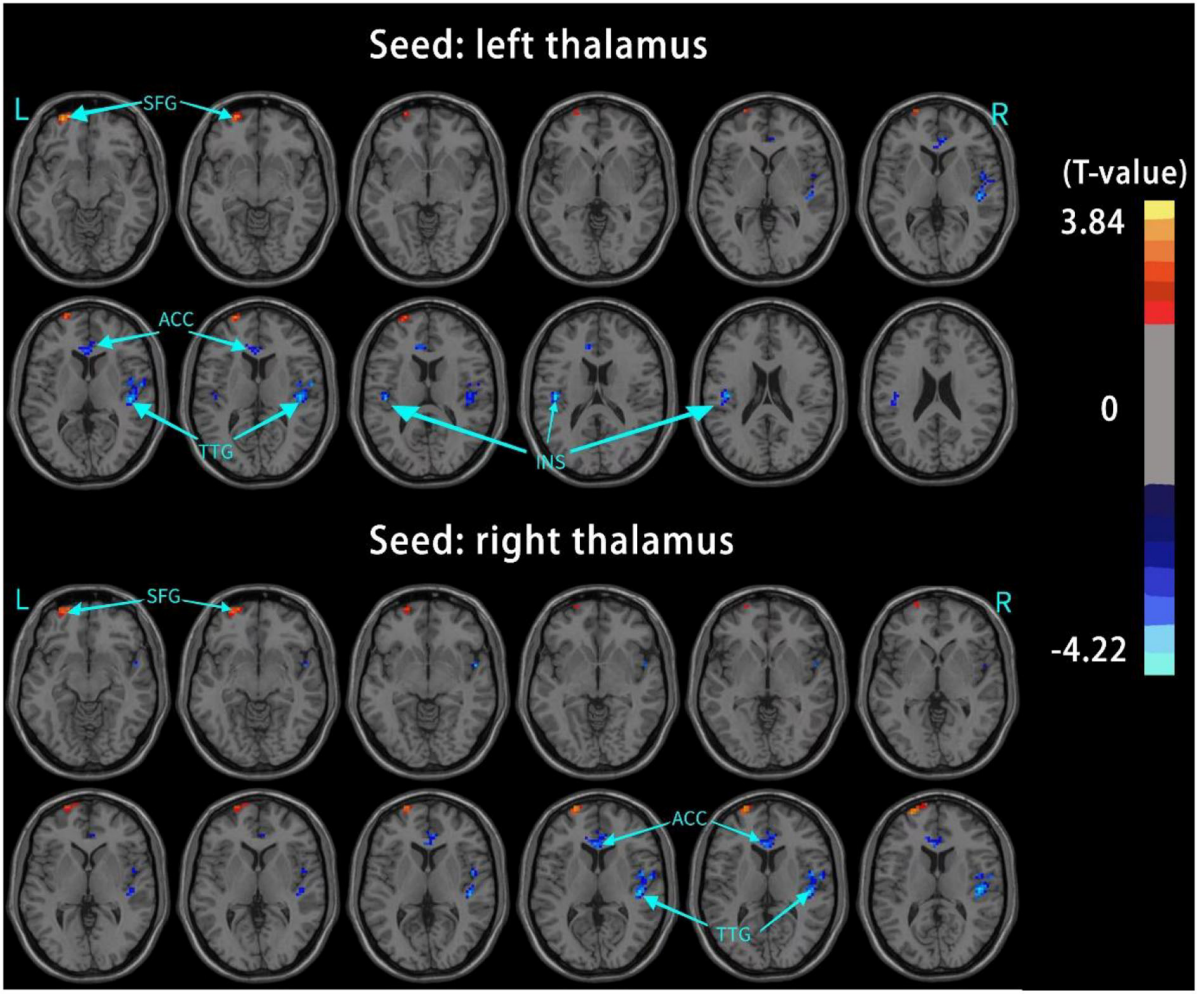
Brain regions with significant changes in the rs-FC of the bilateral thalamus (voxel-level *P* < 0.05, AlphaSim corrected), visualized with the DPABI slice viewer (http://rfmri.org/dpabi). Significant group differences in the mean weighted rs-FC in the above regions (*P* < 0.05). TTG, transverse temporal gyrus; ACC, anterior cingulate cortex; INS, insula; SFG, superior frontal gyrus.

**Figure 2:**
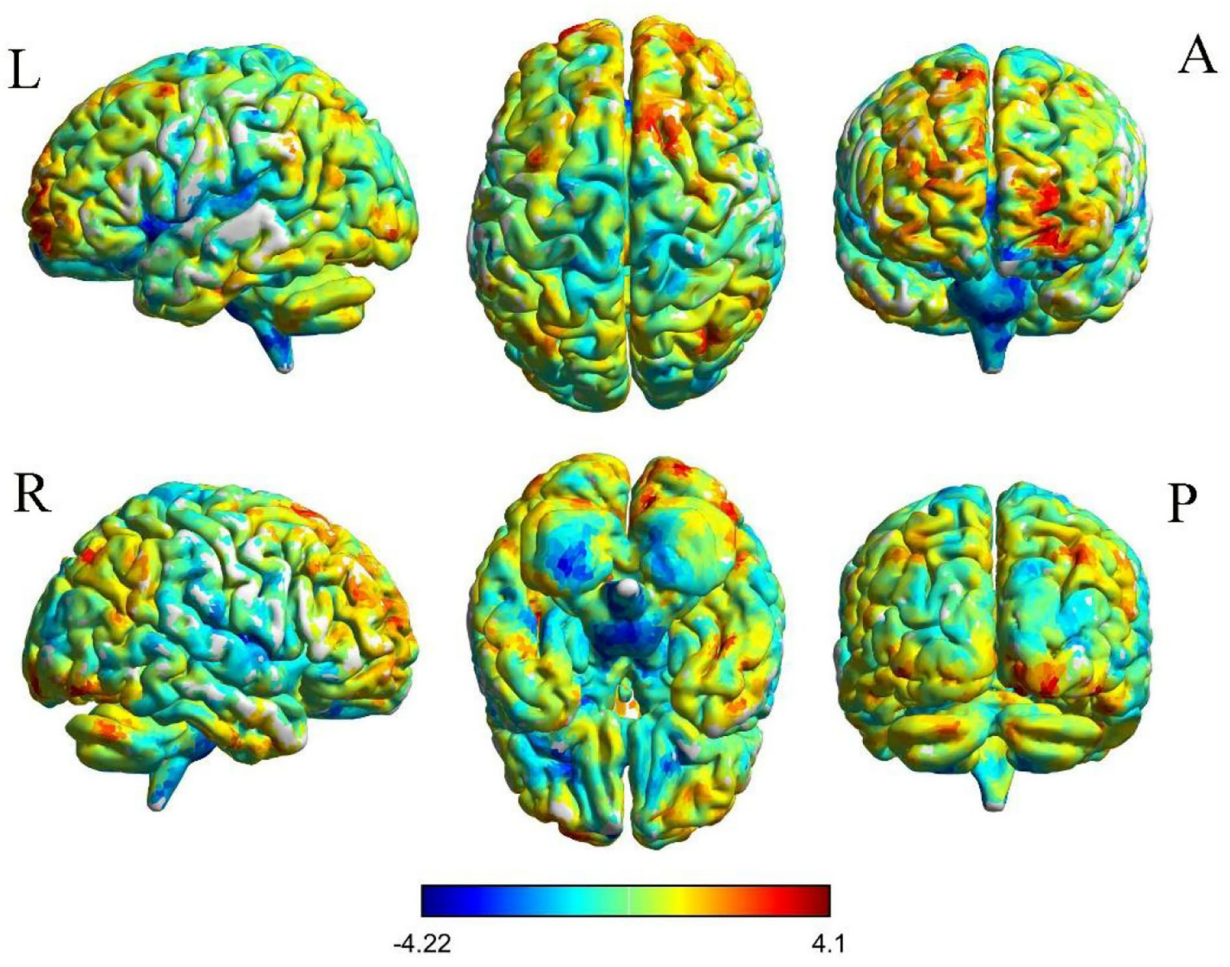
Voxelwise comparison of FC between participants in the PACG and HC groups based on the left thalamus seed (voxel-level *P* < 0.05, AlphaSim corrected). Color bars indicate t-scores; warm colors indicate areas where the FC value in PACG is greater than that in HCs, while cold colors indicate the opposite.

**Figure 3:**
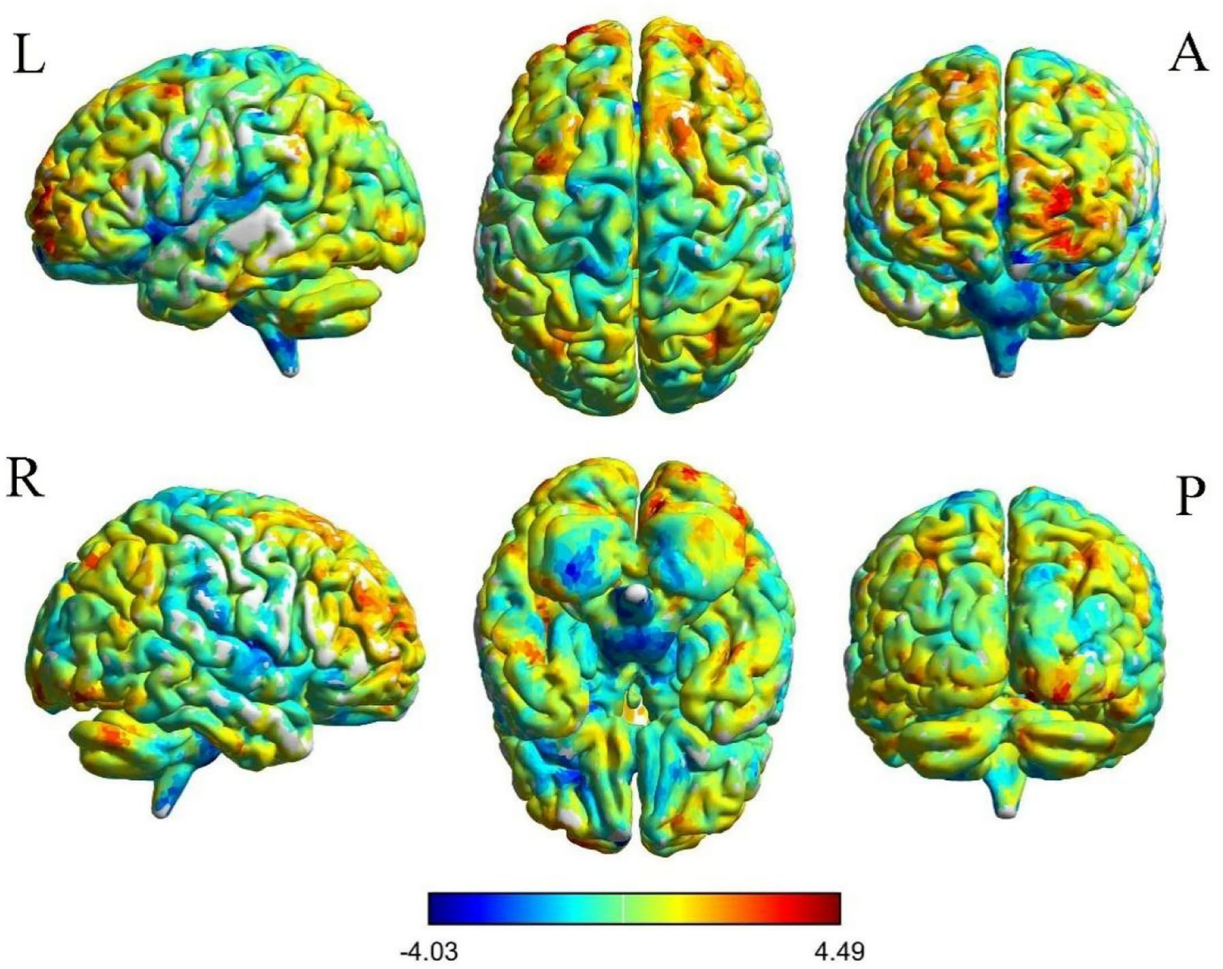
Voxelwise comparison of FC between participants in the PACG and HC groups based on the right thalamus seed (voxel-level *P* < 0.05, AlphaSim corrected). Color bars indicate t-scores; warm colors indicate areas where the FC value in PACG is greater than that in HCs, while cold colors indicate the opposite.

**Figure 4:**
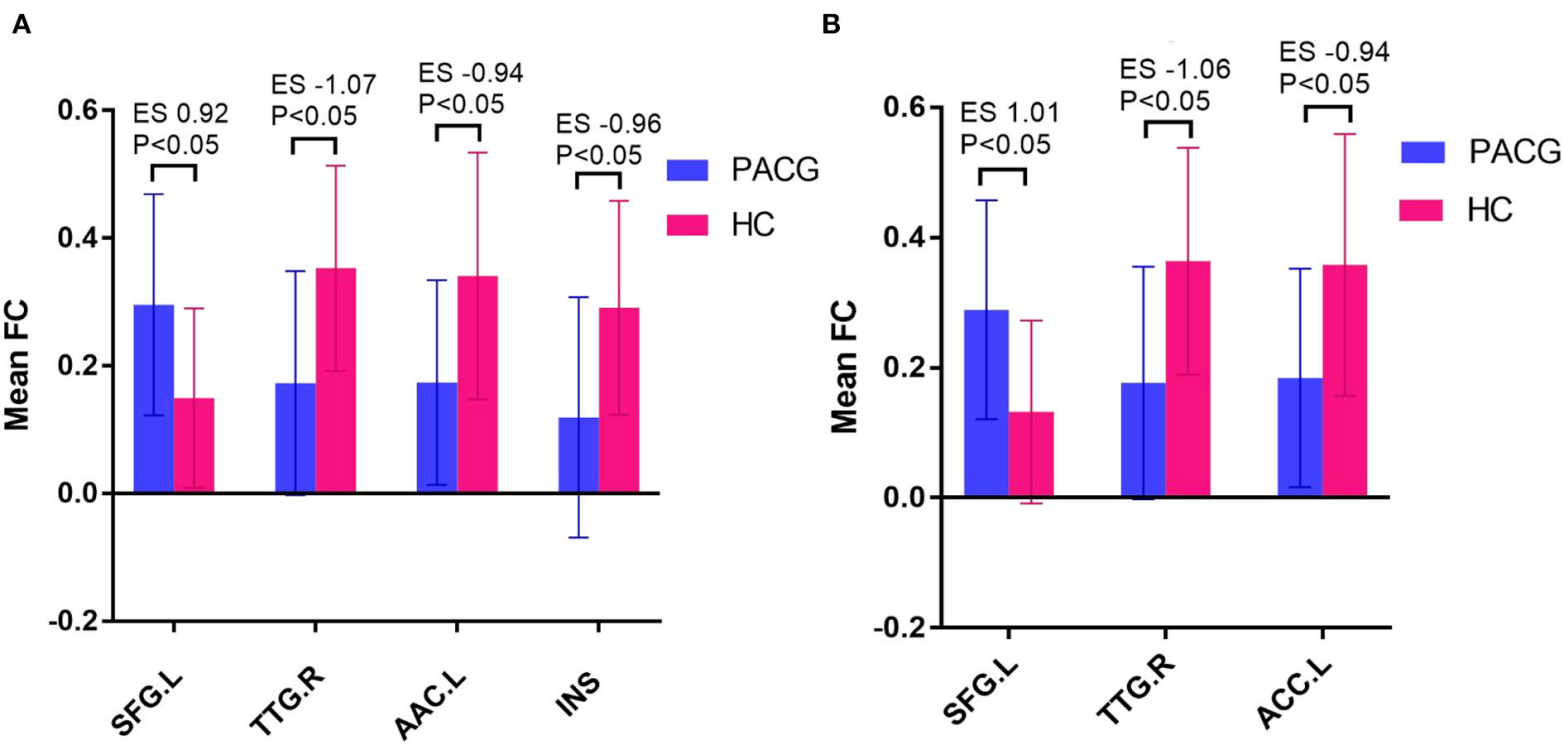
Mean weighted FC values of HCs and PACG patients in altered regional brain areas based on the left thalamus seed **(A)**.and right thalamus seed **(B)**. FC, functional connectivity; PACG, primary angle-closure glaucoma; HCs, healthy controls; TTG, transverse temporal gyrus; ACC, anterior cingulate cortex; INS, insula; SFG, superior frontal gyrus; ES, effect size; L, Left; R, Right.

The results of intergroup rs-FC comparisons at the voxel-based whole-brain level based on bilateral thalamic seeds are displayed in Table 2. Compared with the HCs, the PACG patients showed decreased FC between the bilateral thalamus and right transverse temporal gyrus (TTG), between the bilateral thalamus and left anterior cingulate cortex (ACC), and between the left thalamus and left insular (INS). Concurrently, increased FC was found between the bilateral thalamus and left superior frontal gyrus (SFG) (Figures 1–3). Interestingly, either the decreased or increased FC values of all the above regions were lower or higher than the FC values of the corresponding brain regions in the HC groups (Figure 4).

**Table 2:** Brain areas showing functional connectivity differences with the thalamus between PACG patients and HCs.

		**Brain area**	**Voxel**	**MNI coordinates of peak voxel**			***t*-value**	**ES**
**Seed-ROIs**	**L/R**			***X***	***Y***	***Z***		
Left thalamus
	R	TTG	102	45	−21	9	−4.2158	−1.07
	L	ACC	45	−12	36	15	−3.2792	−0.94
	L	INS	42	−45	−18	18	−4.0631	−0.96
	L	SFG	51	−27	60	−9	3.669	−0.92
Right thalamus
	R	TTG	101	45	−21	9	−4.0337	−1.06
	L	ACC	50	−3	30	9	−3.3854	−0.94
	L	SFG	64	−24	66	12	3.8375	−1.01

Voxel level P < 0.05, AlphaSim corrected. PACG, primary angle-closure glaucoma; HCs, healthy controls; TTG, transverse temporal gyrus; ACC, anterior cingulate cortex; INS, insula; SFG, superior frontal gyrus; ES, effect size.

### Relationships between clinical DATD FC of the thalamus in PACG patients

The correlations between the clinical data and FC values of the PACG patients are summarized in Table 3. The FC of the bilateral thalamus and left SFG in PACG patients was positively correlated with RNFLT (left *r* = 0.432, *P* = 0.011; right *r* = 0.377, *P* = 0.028) but negatively correlated with A-C/D and V-C/D (left A-C/D: *r* = −0.361, *P* = 0.036; left V-C/D: *r* = −0.389, *P* = 0.023; right A-C/D: *r* = −0.345, *P* = 0.045; right V-C/D: *r* = −0.366, *P* = 0.033). The FC between the left thalamus and left INS was negatively correlated with IOP (*r* = −0.444, *P* = 0.008).

**Table 3:** Significant associations between thalamic FC and clinical characteristics in PACG patients.

**FC between brain regions**	**Clinical characteristic**	***r* values**	***P*-values**
Left thalamus–left SFG	RNFLT	0.432	0.011
Right thalamus–left SFG	RNFLT	0.377	0.028
Left thalamus–left SFG	A-C/D	−0.361	0.036
Right thalamus–left SFG	A-C/D	−0.345	0.045
Left thalamus–left SFG	V-C/D	−0.389	0.023
Right thalamus–left SFG	V-C/D	−0.366	0.033
Left thalamus–left INS	IOP	−0.444	0.008

FC, functional connectivity; PACG, primary angle-closure glaucoma; SFG, superior frontal gyrus; INS, insula; RNFLT, retinal nerve fiber layer thickness; A-C/D, average cup-to-disc ratio; V-C/D, vertical cup-to-disc ratio; IOP, intraocular pressure.

## Discussion

The main finding of this study is that PACG patients show extensive abnormalities in FC among multiple brain regions, including regions involved in vision, hearing, emotion, and cognition, which is consistent with our hypothesis.

In our current study, we noticed decreased FC of the bilateral thalamus with the right TTG and the left ACC in PACG patients. The TTG is located in the primary auditory cortex in the lateral sulcus of the brain and is the first cortical structure to receive auditory information, indicating that the auditory function of PACG patients was impaired. Both the auditory cortex and the ACC belong to the frontotemporal network which has been shown to be involved in auditory information processing for cognitive purposes (34). Many studies (35, 36) have found that glaucoma patients have structural and functional abnormalities in the projections from the thalamus to the higher visual cortices, but the connections with the auditory cortex are rarely studied, likely because glaucoma patients mainly present with ocular symptoms rather than hearing impairment. The decreased connectivity between the thalamus and auditory cortex in patients with major depression may lead to mismatched processing at the cortical level, contributing to these patients' clinical symptoms (37). In addition, the ALFF value in the ACC is significantly reduced in patients with end-stage renal disease (ESRD) and is positively correlated with Montreal Cognitive Assessment (MoCA) scores, suggesting that the ALFF value in this region may be an imaging indicator of cognitive dysfunction (38). Thus, we speculated that the PACG might lead to the reorganization of auditory and cognitive function. Similar results that no disruption of measures were also observed in a previous study regarding glaucoma patients (39–41), hinting that the efficiency of communication in the brain network was conserved and the brain was homogeneously well reorganized.

In addition, the INS cortex is extensively connected to the frontal, temporal, parietal, occipital, and limbic regions and participates in multiple processes, such as emotional, cognitive and motor sensory information processing (42). Many previous studies (2, 43) found abnormalities in the structure and function of the INS in glaucoma patients. For example, Chen et al. (16) found that the ReHo value of the right INS in PACG patients was elevated, and Chen et al. (21) found that short-range functional connectivity density (FCD) was increased in the left INS of PACG patients and negatively correlated with the A-C/D. In addition, Li et al. (35) found that preoperative PACG patients had lower FC in the left primary visual cortex and left INS than controls, which is consistent with the results of our study. Clinically, many PACG patients present with anxiety or even depression (44–46). A study (47) showed that the FC of the INS in patients with schizophrenia was generally decreased. Therefore, the decreased rs-fMRI FC between the thalamus and INS in PACG patients may be due to impaired emotional cognitive regulation pathways. However, from the results, the individual differences within the PACG group are relatively large, and a generalizable result may not be drawn. However, this result is not highly reliable because of the large individual variability within the group. The current study cannot explain this result sufficiently, but we will focus on its causes in future research.

The correlation analysis showed that the FC of the left thalamus and left INS in PACG patients was negatively correlated with IOP. Therefore, we speculate that elevated IOP leads to emotional disturbances in glaucoma patients, which would, in turn, lead to dysfunction of the INS.

The left SFG is located above the orbit and is mainly involved in eye movement, visual positioning, advanced cognition and working memory functions (48, 49). Neuroimaging studies (50) have identified functional interactions between the thalamus and the default-mode network (DMN), and the brain regions with increased FC (the SFG) and decreased FC (the ACC) both belong to the DMN. Owing to the death of retinal ganglion cells in PACG patients, the transmission of visual information in PACG patients is blocked, which results in decreased FC between the thalamus and DMN nodes (such as the ACC). To compensate for the decreased FC between the networks, the thalamus and other regions of the DMN (such as the SFG) have increased FC. Many diseases damage the brain regions associated with the DMN, which consumes a great deal of energy in the resting state and is especially susceptible to oxidative stress and disease. For example, Wang et al. (51) found that the FC between the DMN and the visual network was decreased in POAG patients and was positively correlated with the average visual field. Additionally, a previous application of the degree centrality (DC) analysis method to this topic by our group, showed that the DC value of a DMN brain region (ACC) increased in PACG patients (52), indicating that glaucoma patients do have abnormal DMN function.

Furthermore, other factors, such as local neuroplasticity, may play a compensatory role during the progression of glaucoma. Neuroplasticity is a selective phenomenon that functionally and structurally molds synaptic connections of the nervous system through experience (53). Several research (54, 55) have found a perceptual boost in the responses of the deprived eye following short-term monocular deprivation in amblyopic patients. Similar results were also observed in glaucoma patients (56). For patients with glaucoma, the decreasing function of visual cortex, which may compensatory increase the function of other brain regions.

The FC between the bilateral thalamus and the left SFG was positively correlated with the RNFLT, and the RNFLT was associated with the progression of glaucoma. Therefore, we speculate that the progression of glaucoma may be accompanied by cognitive impairment. However, the FC between the bilateral thalamus and left SFG was negatively correlated with A-C/D and V-C/D, while A-C/D and V-C/D were positively correlated with the incidence of glaucoma and impairment of the anterior visual pathway (57), indicating that the brain's compensatory action cannot reverse the development of glaucoma and damage to the eye.

## Limitations

There are several limitations to our study. First, the patients' emotions were not measured. PACG patients have been reported to be more sensitive to negative affective states, such as depression and anxiety, than HCs or even POAG patients. Detailed psychological scales should be included in any further research on this topic. Second, longitudinal research is lacking. The duration glaucoma disease varies greatly, and so the specific temporal relationship between brain activity alterations and disease stage is still unclear.

## Conclusion

In conclusion, there are extensive abnormalities in resting-state FC between the thalamus and vision, hearing, emotion, and cognition related brain regions in PACG patients, and these differences are correlated with clinical variables, which suggests that abnormal FC of the thalamus plays an important role in the progression of PACG.

## Data availability statement

The raw data supporting the conclusions of this article will be made available by the authors, without undue reservation.

## Ethics statement

The studies involving human participants were reviewed and approved by the Medical Research Ethics Committee of The First Affiliated Hospital of Nanchang University. The patients/participants provided their written informed consent to participate in this study.

## Author contributions

XZ and LC guided and designed the MRI experiment, besides, they reviewed and revised the manuscript. FC and YW analyzed the resting-state fMRI data. YW unscrambled the results and wrote the manuscript. JG, FO, YC, and MY collected resting fMRI data and applied for the ethics. CH helped with statistics and graphing. All authors contributed to the article and approved the submitted version.

## Funding

This study was supported by the National Natural Science Foundation of China (Grant No. 81760307) and the Natural Science Foundation Project of Jiangxi, China (Grant No. S2019ZRZDB0311).

## Conflict of interest

The authors declare that the research was conducted in the absence of any commercial or financial relationships that could be construed as a potential conflict of interest.

## Publisher's note

All claims expressed in this article are solely those of the authors and do not necessarily represent those of their affiliated organizations, or those of the publisher, the editors and the reviewers. Any product that may be evaluated in this article, or claim that may be made by its manufacturer, is not guaranteed or endorsed by the publisher.
